# Moving towards universal health coverage: engaging non-state providers

**DOI:** 10.1186/s12939-018-0844-7

**Published:** 2018-10-05

**Authors:** Zubin Cyrus Shroff, Krishna Dipankar Rao, Sara Bennett, Ligia Paina, Marie-Gloriose Ingabire, Abdul Ghaffar

**Affiliations:** 1 Alliance for Health Policy and Systems Research, World Health Organization, Geneva, Switzerland; 2 Johns Hopkins University Bloomberg School of Public Health, Baltimore, USA; 3 International Development Research Centre, Ottawa, Canada

**Keywords:** Universal health coverage, Non-state providers, Contracting, Low-and-middle-income countries

## Abstract

This editorial provides an overview of the special issue “Moving towards UHC: engaging non-state providers”. It begins by describing the rationale underlying the Alliance’s choice of a research program addressing issues of non-state providers and briefly discusses the research process this entailed. This is followed by a summary of the findings and key messages of each of the eight articles included in the issue. The editorial concludes with a series of reflections regarding lessons learnt about the engagement of non-state providers, methodological challenges, areas for future research as well as the contribution of the research program towards efforts to build capacity and strengthen health systems towards universal health coverage.

## Introduction

The rise of universal health coverage (UHC) to the top of the global health agenda has brought renewed attention to the role of the heterogenous group of non-state providers (NSPs) to deliver services and contribute to the achievement of public health goals. In many low-and-middle income countries (LMICs), the challenge of effectively delivering quality health services to all who need them is ever more apparent, as is the realization that all available human resources for health, whether in the public or private sector, need to be engaged to achieve this goal. Coupled with an increasing recognition of the major, and often dominant, role of NSPs in service delivery in LMIC settings, this has spurred governments to engage with NSPs through a range of interventions. These include contracting, social marketing and providing training among others [1–3].

Proponents of formal government engagement with NSPs argue that they operate widely, even in remote and rural areas, and that patients perceive them to be more responsive than their public sector counterparts [4]. Further, through formal engagement, governments can hold NSPs accountable for meeting standards and achieving results, hence improving the quality of their services [5]. However, evidence of the impact of engaging NSPs on utilization and quality of services, as well as on out-of-pocket health expenditures in LMICs is mixed. Some studies report notable increases in service coverage, utilization and/or quality of care, while others report slight or weak effects [2, 6–9]. If the evidence on impact is mixed, even less is known about why and how governments’ interventions to engage NSPs succeed (or not). Building the evidence around government engagement of NSPs is, therefore, both timely and essential for the effective design and implementation of policies and programs and for meeting public health goals.

The need for new knowledge in this area was identified through a consultative priority-setting process supported by the Alliance for Health Policy and Systems Research (HPSR) that sought to identify priority research questions relating to NSPs. This consultative process included: key informant interviews with health policymakers, researchers, and community and civil society representatives across 24 LMICs around the world; a review of existing literature; and a stakeholder consultation held in Bellagio, Italy, that solicited input from nine research experts well-versed in this area [10].

## Research process

Informed by the findings of the priority-setting process and aware of the potential for new knowledge in this area to inform ongoing efforts to strengthen health systems towards UHC, the Alliance for HPSR with support from Canada’s International Development Research Centre (IDRC) and the Rockefeller Foundation announced a call for research in June 2014. Researchers based in LMICs were invited to submit proposals to develop analytical case studies to explain the performance (including successes and failures) of interventions to engage NSPs in strengthening health systems moving towards UHC. For the purpose of the call, non-state providers were defined broadly in line with Palmer [11] and Mills et al. [12]^1^ and relevant interventions of interest included regulatory and policy changes, contracting, financing, social marketing and training.

Seventy-seven proposals were received in response to the call; after an extensive review process, eight projects were selected for funding. The selection of these cases was based primarily on the merit of the research proposal, but also to ensure diversity in both country context, and the nature of private sector engagement. The selected research proposals came from teams in Afghanistan, Bangladesh, Bosnia-Herzegovina, Burkina Faso, Ghana, South Africa, Tanzania and Uganda (Table 1). A team from the Johns Hopkins Bloomberg School of Public Health provided ongoing technical support to the project teams, including on developing protocols and research tools, conducting data analysis and scientific writing.

**Table 1:** List of selected projects

Country studied	Title
Afghanistan	A case study of the Basic Package of Health Services (BPHS) in Afghanistan
Bangladesh	Urban primary health care in Lower Middle-Income Countries - is contracting out the answer?
Bosnia-Herzegovina	Engaging private health care providers in implementation of mandatory safety standards in the Republic of Srpska
Burkina Faso	A public private partnership for health systems strengthening: a case study of scaling up community based management of malaria in Burkina Faso
Ghana	Slow Systems Integration Towards Universal Health Coverage: Strengthening the relationship between faith-based non-profit providers and the Ghanaian state system
South Africa	Case Study on the Role of General Practitioner Contracting in strengthening Health Systems towards Universal Health Coverage in South Africa
Tanzania	Engaging non-state providers towards Universal Health Coverage: The impact of contracting-out on health services and health outcomes at the District Level in Tanzania
Uganda	The Role of Government Subsidies to Non-Profit Health Providers in Extending Universal Health Coverage Goals in Uganda

## Findings

This collection, comprising seven country studies and one cross-country analysis,^2^ begins with two papers that examine experiences of governments contracting with non-governmental organizations (NGOs) in post-conflict Afghanistan and urban Bangladesh. These are followed by three papers that use a range of methods, including causal loop diagrams, geospatial mapping and historical analysis, to shed light on the engagement of faith-based NSPs in three sub-Saharan countries, Tanzania, Uganda and Ghana. The next paper discusses South Africa’s experience in developing and managing context-appropriate contracting-in models to engage physicians in the provision of primary health care. The country studies conclude with a paper from Bosnia and Herzegovina that brings to the fore some of the challenges in effectively implementing regulation of for-profit providers. Finally, based on learnings from the country papers, Rao et al. [5] derive new lessons for effective contracting of NSPs in LMICs by governments keen to explore ways to move more efficiently and effectively towards UHC.

The first paper, by Salehi et al. [13] examines the contextual, contractual and institutional factors influencing the performance of NSPs contracted to provide services under Afghanistan’s Basic Package of Health Services (BPHS). Difficult geography, socio-cultural influences such as the need for female health care providers to serve female clients, the lack of basic security, high staff turnover, and political interference in hiring and firing decisions were cited as some of the factors that negatively influenced performance. On the other hand, having in place well-defined and formal contracts as well as supportive political leadership at the provincial level were cited as positive influences. At an institutional level, contracting out was credited with spurring the development of an effective national health management information system (HMIS), the development of capacity among NSPs to successfully serve as contractors for the BPHS and strengthening the capacity of the Ministry of Public Health to manage the contracting-out process. The paper concludes with observations on the need for the contracting-out process to better take into account contextual differences among provinces and the potential of engaging for-profit providers as well in the provision of services under the BPHS.

In the second paper in the collection, Islam et al. [14] explore the contextual, contractual and actor-related factors influencing the evolution of contracting-out for urban primary health care in Bangladesh, a process that has undergone three phases over more than two decades. The choice of the Ministry of Local Government, as opposed to the MOH, as the implementing agency, non-alignment with pre-existing public programs with similar mandates, political interference in selection of project areas and staffing decisions, and the difficulty of staff retention in the face of increased pay-scales in the public sector were some of the barriers to effective implementation. Effective implementation was also hampered by the non-alignment of program objectives with contracting rules and processes. For example, cost-recovery targets conflicted with serving the poorest members of the population, and quality problems arose from awarding contracts to the lowest bidder without due consideration of the technical quality of proposals. The authors argue for more effectively aligning contracting-out objectives with policies and guidelines, developing in-country contracting-out capacity and building partnership-oriented relationships between governments and NSPs to replace the often-hierarchical relationships that have hampered effective collaboration.

The next three papers examine the engagement of faith-based providers in the provision of health services in Tanzania, Uganda and Ghana. Maluka et al. [15] examine factors influencing the design and implementation of Service Agreements between local governments and faith-based providers in Tanzania. This contractual modality marked a definitive shift from collaborations built on informal trust-based relationships to a system backed by legal frameworks. The paper finds that the development of Service Agreements was enabled by technical and financial support from donors. While districts were technically empowered to establish contracts directly with NSPs, their financial dependence on donor funds channeled through the central government limited their actual autonomy. Delayed reimbursements, inadequate administrative capacity especially at the local government level, and the absence of a mechanism to resolve disagreements negatively influenced contract implementation. This paper highlights the need for strengthening capacity within both governments and NSPs to develop and manage contracts, the importance of effective monitoring, and the need for ongoing communication to clarify expectations and resolve any misunderstandings among the various stakeholders.

The next paper by Ssennyonjo et al. [16] analyzes trends in Government Resource Contributions (GRCs) to NSPs by examining the case of primary healthcare (PHC) grants made by the Ugandan government to the Ugandan Catholic Medical Bureau (UCMB) between 1997 and 2015. The authors use a complex adaptive systems framework to explain changes in contributions and the evolution of the relationship between the government and UCMB over time. They identify three phases in the evolution of the grants: an early initiation phase (1997–2000), a phase of rapid increase (2000–2005), and a phase of decline (2005–2015). These phases were influenced by the availability of public funding, broader donor funding modalities and attitudes of government leaders regarding the private not-for-profit (PNFP) sector. Using a series of causal loop diagrams, the authors demonstrate the complex responses engendered by the changing dynamics in each phase, including changes in user fees, altered expectations of UCMB on the part of the government, professionalization of UCMB services, increasing transparency and information sharing and efforts by UCMB to determine the costs of providing services.

In the third paper on faith-based NSPs, Grieve and Olivier [17] map the development of the faith-based non-profit health sector in Ghana’s health system over more than five decades. Bringing together qualitative, quantitative and geospatial data, along with various documentary sources, they developed geospatial maps to provide a visual representation of the changing distribution of facilities affiliated to the Christian Health Association of Ghana (CHAG), a leader in the Ghanaian faith-based not-for-profit health sector. In line with CHAG’s founding mission to serve the poorest and most marginalized, these facilities were originally largely located in remote, rural parts of Ghana. However, this has changed over time. The urbanization of areas once regarded as remote and rural (often because they were the sites of facilities such as Mission Hospitals) and the expansion of the public health sector into areas once served exclusively by faith-based providers, has reduced NSP’s prioritization of access for the rural poor. The paper concludes by observing the potential of utilizing tools such as geospatial mapping in identifying gaps and duplications in services to enable the most effective use of resources for the health system as a whole.

The sixth article in the collection, by Mureithi et al. [18], examines policy processes underlying the evolution and emergence of three contracting models under South Africa’s General Practitioner Contracting Initiative (GPCI) pilot. This contracting-in mechanism to address public sector physician shortfalls was piloted as part of the country’s National Health Insurance program. The study found that, although funded from a single source, different contracting models emerged during the period 2011–2014. The differences, based on the type of purchaser and with different levels of involvement  of  national, provincial and district actors, developed through an iterative process into three distinct models: the centralized-purchaser model, the decentralized-purchaser model and the contracted-purchaser model. Financial management capacity, managerial capacity and the ability to innovate all influenced the development of different models suited to differing contexts. Based on evidence obtained through key informant interviews, focus group discussions and document review, the authors make a case for contracting mechanisms that combine well-defined contract specifications with enough flexibility that local-level adaptation for effective implementation remains possible.

The seventh paper, a study by Rakic et al. [19] on Bosnia-Herzegovina, sheds light on some of the challenges entailed in regulating NSPs in a setting characterized by limited government enforcement capacity. They describe a mechanism designed to certify provider compliance with mandatory safety and quality standards, which has been in place since 2012, and examine why providers do or do not adopt. The authors found the rate of certification differed by type of private provider, comparing dentists, specialists and pharmacists. Using diffusion of innovation theory to explain differences in the rate of adoption of the standards, the study finds that important determinants of the decision whether to certify include concern about being fined or losing a contract with the National Health Insurance Fund, the availability of information on the standards and accreditation process and the relevant professional association’s level of support for the introduction of standards. The paper concludes that providing information, establishing a system of incentives and penalties and closely engaging with professional associations are all needed to encourage all providers to seek certification of compliance with mandatory standards.

Based on the findings from the country studies, the final paper in this collection provides cross-cutting lessons on contracting NSPs in LMIC settings to move towards UHC. Rao et al. [5] observe that governments contract with NSPs for the delivery of health services for a variety of reasons, including weak capacity and a shortage of human resources in the public sector and a large pool of non-state providers. However, the authors note that contracting NSPs has not, on its own, overcome major service delivery challenges, including attracting and retaining health workers. Second, the institutional capacity of all actors involved in the contracting process at national, sub-national and local levels greatly influences the success of contracting. Governments and NSPs alike require sufficient human, financial, monitoring and administrative capacity to effectively develop and manage contracts; they also require the flexibility to adapt to contextual differences and changes over time. Third, developing and managing good relationships between governments and NSPs was found to be a key to long-term success with contracting health services. Finally, government stewardship capacity, including to effectively enforce regulation and minimize political interference in contract implementation, has an important bearing on the success of contracting.

## Conclusion

This special issue makes an important contribution to understanding how governments can effectively engage NSPs to strengthen health systems towards UHC. The eight papers provide valuable learning from experiences that reflect significant diversity on various axes: the level of socio-economic development (from post-conflict, low-income Afghanistan to upper-middle income Bosnia-Hercegovina and South Africa); the type of NSP engaged (including NGOs, faith-based providers and the for-profit sector); the means of engagement (contracting and regulation); and the methods and tools used (including descriptive and analytical case studies, causal loop diagrams and geospatial mapping).

Despite its diversity, this collection also reveals surprising similarities among the cases examined. As Rao et al. [5] argue in the cross-country analysis, all governments face common challenges: building institutional capacity to manage NSP engagement; effectively enforcing rules and regulations; minimizing inappropriate political interference in key decisions; ensuring the quality of health services provided; and developing and maintaining trusting relationships among all the contracted parties and other stakeholders to facilitate effective collaborations.

The seven country-specific papers also highlight the value of conducting in-depth study of implementation processes. This focus provides a critically-needed complement to other findings on the impact of engaging NSPs in health services delivery. These deep examinations were made possible by integrating findings from both quantitative and qualitative research methods. Employing qualitative methods and tools enabled the research teams to address the questions of “why and how did this happen?” raised by quantitative analyses of “what happened?”

The papers also highlight some methodological challenges, in particular the difficulty of using retrospective interviews to build accurate and coherent narratives and explanations. Improving real-time documentation of policy processes for NSP engagement could significantly improve our ability to analyze and understand these processes; this, in turn, would lead to improved policy design and implementation.

Reading the papers in the collection together also highlights a key tension inherent in any social development effort: balancing the long-term commitment required to promote program stability and the strength of the stakeholder relationships with the importance of establishing and enforcing clear performance targets, incentives and sanctions to ensure optimal results.

While the final paper in the collection provides insightful lessons, it also points to some outstanding needs. First, there are relatively few cross-country comparisons and analyses on the role of NSPs, and additional work of this nature could create further transferable, policy-relevant insights. We hope to see more research programs use common frameworks to examine similar policy development and intervention processes across different settings. This approach enables researchers to make powerful inferences, while preserving the depth of detail that a single case study provides. We also hope that future research efforts will examine individual policy processes using multiple theoretical frameworks. From a methodological point of view, this would contribute both to refining the theoretical basis for related research and to identifying effective research methods for this arena. In terms of the topics covered by this supplement, it is notable that all of the interventions studied engagement with formal rather than informal health care providers, whereas in many parts of the world, the poor, in particular, rely heavily on informal health care [20]. There was also a preponderance of studies on contracting-out mechanisms, whereas incentive based strategies to improve quality of private sector care (such as accreditation), but also regulation, and public/private partnerships involving capital investments are not as well represented in this supplement, nor in the broader literature. Given the diversity of private health care providers, as well as instruments to engage with them, much more work in this field is warranted.

This special issue—and the research program that supported its development— exemplifies the unique value added by the Alliance for Health Policy and Systems Research to the landscape of research on health systems. In this case, the Alliance identified an under-researched health systems topic and catalyzed the generation of new knowledge to fill critical gaps and inform future policies, while also strengthening the capacity of research teams. This effort played out over nearly four years and involved intensive engagement with research teams in eight countries, some of which are relatively new to the field of health policy and systems research. In making and sustaining these connections, the Alliance has hopefully enlarged the community of health systems researchers, in particular those actively studying the engagement of NSPs in contributing to public health goals.

Finally, this research programme would not have been possible without the collaboration and support of the International Development Research Centre, Canada, and the Rockefeller Foundation. This support reflects a far-sightedness and commitment to investing in the development of the capacity of researchers in LMICs, which is essential to strengthening health systems to support UHC. The partners and researchers who collaborated on this research program hope that its findings will serve as a further catalyst, sparking new or renewed interest among policymakers to engage with NSPs in the achievement of public health goals and among health systems researchers to delve ever-deeper into understanding how these efforts become successful.

## Abbreviations

BPHS: Basic Package of Health Services
CHAG: Christian Health Association of Ghana
HPSR: health policy and systems research
IDRC: International Development Research Centre
LMICs: low and middle-income countries
NGOs: non-governmental organizations
NSPs: non-state providers
UCMB: Ugandan Catholic Medical Bureau
UHC: universal health coverage

## Footnotes

1 “All providers who exist outside the public sector, whether their aim is philanthropic or commercial, and whose aim is to treat illness or prevent disease. They include large and small commercial companies, groups of professionals such as doctors, national and international non-governmental organizations, and individual providers and shopkeepers. The services they provide include hospitals, nursing and maternity homes, clinics run by doctors, nurses, midwives and paramedical workers, diagnostic facilities such as laboratories and radiology units, and the sale of drugs from pharmacies and unqualified static and itinerant (mobile) drug sellers including general stores.”

2 The results from the project in Burkina Faso are being submitted in a French language journal and are hence not included in this collection
